# Impact of persistence to secondary preventive medication on prognosis for patients with myocardial infarction with and without obstructive coronary arteries

**DOI:** 10.1371/journal.pone.0324533

**Published:** 2025-05-29

**Authors:** Anna M. Nordenskjöld, Lars Lindhagen, Björn Wettermark, Bertil Lindahl

**Affiliations:** 1 Department of Cardiology, Faculty of Medicine and Health, Örebro University, Örebro, Sweden; 2 Uppsala Clinical Research Center, Uppsala University, Uppsala, Sweden; 3 Department of Pharmacy, Faculty of Pharmacy, Uppsala University, Uppsala, Sweden; 4 Department of Medical Sciences, Uppsala University, Uppsala, Sweden; Saud Al-Babtain Cardiac Centre, SAUDI ARABIA

## Abstract

**Background:**

Poor adherence to secondary preventive medication after myocardial infarction (MI) negatively affects long-term prognosis, but knowledge is lacking regarding the impact of poor adherence on prognosis for patients with myocardial infarction with non-obstructive coronary arteries (MINOCA). We therefore investigated the effect of persistence to secondary preventive medication on prognosis in patients with MINOCA compared with patients with myocardial infarction with obstructive coronary arteries (MI-CAD).

**Methods:**

In this nationwide observational study of 116,143 patients with MI recorded in the SWEDEHEART registry between 2006─2017, MINOCA were identified in 9,124 patients and MI-CAD in 107,019 patients. Persistence to treatment with aspirin, statins, beta blockers and angiotensin-converting enzyme inhibitors (ACEIs) or angiotensin-receptor blockers (ARBs) was investigated for 5 years post discharge and patients were followed for a composite endpoint of major adverse cardiovascular events (MACE), including all-cause death, MI, ischemic stroke and heart failure.

**Results:**

Persistent use of secondary preventive medications was associated with a decrease in the risk of MACE during follow-up in both MINOCA and MI-CAD patients; aspirin HR 0.70 (CI 0.60–0.82) vs. HR 0.60 (CI 0.57–0.64), statins HR 0.80 (CI 0.68–0.95) vs. HR 0.66 (CI 0.63–0.69), beta blockers HR 0.77 (CI 0.65–0.92) vs. HR 0.76 (CI 0.73–0.80) and ACEIs/ARBs HR 0.62 (CI 0.50–0.77) vs. 0.67 (CI 0.63–0.71).

**Conclusion:**

Persistence to secondary preventive medications after MI is associated with a reduction in the risk for MACE in both patients with MINOCA and MI-CAD. Continuous efforts to improve adherence to evidence-based medications in general to all patients with MI should be a priority.

## Introduction

Cardiovascular disease is the most common cause of morbidity and mortality worldwide [1]. International guidelines recommend secondary preventive treatment, including control of cardiovascular risk factors; lifestyle changes; and secondary preventive medications to improve outcome after acute myocardial infarction (MI) [2,3].

Poor medication adherence to secondary preventive therapy with aspirin, P2Y12-inhibitors, statins, angiotensin-converting enzyme inhibitors (ACEIs)/angiotensin-receptor blockers (ARBs) and beta blockers have repeatedly been demonstrated [4–7] and shown to unfavourably affect the prognosis [4,5,8]. According to a large meta-analysis, approximately 9% of all cardiovascular events in Europe may have been caused by poor medication adherence [4].

Myocardial infarction with non-obstructive coronary arteries (MINOCA) affect approximately 6–8% of all patients with MI [9,10]. Specific secondary preventive strategies for MINOCA were proposed recently by both the European Society of Cardiology (ESC) [11] and the American Heart Association (AHA) [12]. In the guidelines from ESC, patients with MINOCA of unknown cause may be treated according to secondary prevention guidelines for atherosclerotic disease (class IIb recommendation) and followed-up in the same way as patients diagnosed with MI with obstructive coronary arteries (MI-CAD). Hence, antiplatelet agents should be used to reduce risk for thrombotic events, statins to reduce low-density lipoprotein cholesterol levels (LDL-C) to a sufficient level and blood-pressure agents to target age appropriate blood-pressure levels [3,11]. The scientific statement from AHA also recommends that all modifiable risk factors for cardiovascular disease should be treated aggressively in MINOCA patients with any evidence of atherosclerosis [12]. Furthermore, previous observational studies indicate long-term beneficial effects of treatment with secondary preventive medications, achieving target range LDL-C and participation in exercise training programs [13–16].

The aim of the present study was to investigate the association between persistence to secondary preventive medications and long-term prognosis in patients with MINOCA and MI-CAD.

## Methods

In this nationwide, observational, registry-based cohort study all patients diagnosed with acute MI and registered in the Swedish Web-system for Enhancement and Development of Evidence-based care in Heart disease Evaluated According to Recommended Therapies (SWEDEHEART) registry [17] between January 1, 2006 and December 31, 2017, were available for inclusion.

### Data sources

Data from the SWEDEHEART registry were merged with census data (death and migration) for the Swedish population and two Swedish population-based mandatory national registries; the ‘Prescribed Drug Register’, which contains complete data from all pharmacies in the country on drugs dispensed to individual patients [18], and the ‘Patient Register’, which includes all International Classification of Diseases (ICD) codes and procedures for all hospital admissions and ambulatory care consultations in hospitals [19]. The National Board of Health and Welfare warranted the compilation of data that were linked through the unique social security number that all Swedish inhabitants have [20].

Data on prescriptions for the following medications were included: acetylsalicylic acid (ATC-code B01AC06); P2Y12-inhibitors (B01AC04, B01AC22 and B01AC24); statins (C10AA and C10BA); ACEs/ARBs including fixed combinations with thiazides (C09) and beta blockers (C07).

The following secondary preventive measures were collected from the 12-months follow-up reported in the part of SWEDEHEART on secondary prevention: low-density lipoprotein cholesterol (LDL-C), self-reported nonsmoking and participation in exercise training within a cardiac rehabilitation program.

### Patient selection

**Fig 1:**
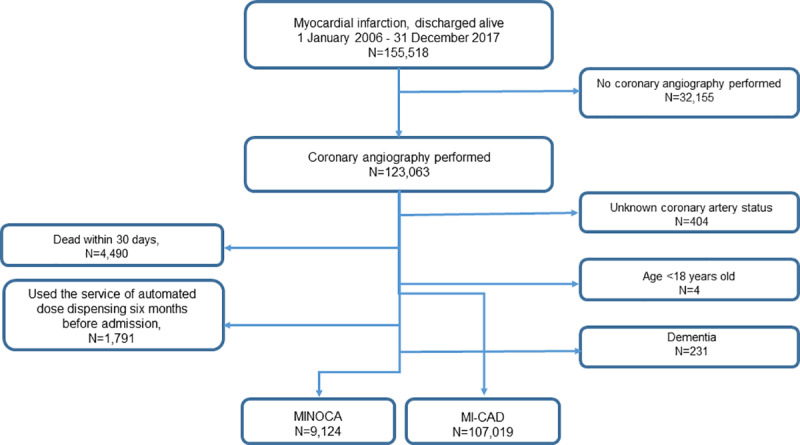
Study population. Patients were excluded if they did not undergo in-hospital diagnostic coronary angiography, if their result of the coronary angiography is unknown, died within 30 days after discharge, were automatically dispensed doses of medication, were < 18 years old or suffered from dementia. Patients with previous PCI or CABG were considered to have a MI-CAD.

Patients were excluded if in-hospital diagnostic coronary angiography was not performed, if the result of the coronary angiography was unknown, death within 30 days after discharge, younger than 18 years, previous diagnose of dementia, or use of automated dose dispensing of medication service before admission. If the coronary angiography performed during the hospitalization showed no stenosis or a diameter stenosis of ≤50% patients were identified as having MINOCA. Patients who had previously undergone percutaneous coronary intervention (PCI) or coronary artery bypass grafting (CABG) were included in the MI-CAD group independently on the findings on latest coronary angiography. The patient cohort consisted of 116,143 individuals where 9,124 were diagnosed as MINOCA and 107,019 were diagnosed as MI-CAD (Fig 1). Patients were followed from hospital discharge to a major adverse cardiovascular event (MACE), including all-cause death, MI, ischemic stroke and heart failure; or until the end of the study period, whichever occurred first. Details regarding the patient selection in this Swedish nationwide register-based cohort study have been published previously [21]. The definitions of the variables constituting the inclusion criteria varied slightly between the former and the present data extraction, therefore the exact number of patients in the MINOCA and MI-CAD groups differs marginally between studies.

According to Swedish law, no written informed consent is required for registration in the SWEDEHEART registry. Information regarding participation, the right to not participate and the opportunity to erase already collected data upon request must be given to all patients.

The study was approved by the Regional Ethical Review Board 2012/60–31/2.

### Exposure

The persistence to medication was only assessed in patients who received their first prescription for a secondary preventive drug at hospital discharge and initiated treatment (filled a prescription for that drug within 30 days after discharge). Patients were regarded as persistent to a drug, if the prescription was refilled within the estimated time of supply from the previous dispensing, including drugs carried over from previous prescriptions and a grace period of 45 days. Patients were allowed to switch between drugs within the same drug class (ATC group). Patients who discontinued treatment were labelled non-persistent.

Patients resuming treatment after being regarded non-persistent were followed as a separate group, labelled “restarters”. The combined group of persistent and restarting patients was labelled “users”. This provided a chance to detect patients resuming treatment after non-persistence and the ability to assess the real proportion of patients with medical treatment at a certain time. A detailed description of the definition of persistence as a construct of adherence, used in this study has been published previously [21].

Patients were evaluated at 2 months, 6 months, 12 months, 2 years, 3 years, and 5 years after hospital discharge. Patients persistent at a certain time period were considered to be persistent until the next pre-defined time period.

### Outcome

Follow-up started at 30 days after discharge and the primary outcome MACE was defined as a composite of all-cause death, re-hospitalization for MI (ICD I21, I22), ischemic stroke (ICD I63, I64) and heart failure (ICD I50, I11.0, I13.0, I13.2). Additional secondary outcomes were all-cause death alone and cardiovascular (CV) death.

### Statistics

Categorical variables were described as frequencies and percentages, and continuous variables were presented as median and interquartile range (IQR). All data sources provided follow-up data until December 31, 2017 except the cause of death registry, where follow-up ended December 31, 2016. Therefore, the CV-death endpoint was only followed until this date.

The association between persistence and clinical outcomes was studied by Cox models. Adjustment was performed for the following potential confounders: age, gender, diabetes mellitus, hypertension, BMI, total cholesterol, cancer, as well as previous MI, heart failure, ischemic stroke, hemorrhagic stroke, and major bleeding. Persistence was added as a time-dependent covariate, as were the covariates age and previous disease. For the adjusted models, a multiplicity correction was applied within the set of four drugs, using the Bonferroni method.

The association between persistence and 1-year targets was studied using logistic regression, adjusting for the same confounders as above, except that all values were fixed at baseline. Hence, there were no time-dependent covariates, and previous MI was not adjusted for.

Missing values of confounders and 1-year targets were handled by multiple imputation (S1 Table). Using the method of chained equations as implemented in the R package mice [22], 10 imputed data sets were generated. All statistical analyses were performed in R, version 4.2.1.

## Results

A total number of 9,124 patients diagnosed with MINOCA and 107,019 diagnosed with MI-CAD were followed-up for a median 5 years. Patients with MINOCA were younger, more often women with fewer risk factors for cardiovascular disease compared to patients with MI-CAD (Table 1).

**Table 1:** Baseline demographic and clinical characteristics of the study population.

	MINOCA	MI-CAD
**Total, n**	9,124	107,019
**Demographics**		
Male, n (%)	3,359 (36.8%)	76,915 (71.9%)
Age, y, median (IQR)	67 (58-74)	68 (59-76)
**Risk factors, n (%)**		
Smoking	1,671 (19.1%)	27,438 (26.6%)
Diabetes	1,275 (14.0%)	2,647 (20.2%)
Hypertension	4,256 (46.6%)	51,632 (48.2%)
BMI kg/m^2^, median (IQR)	26.1 (23.4-29.4)	26.6 (24.3 -29.4)
Heart failure	434 (4.8%)	4,222 (3.9%)
Ischemic stroke	280 (3.1%)	4,232 (4.0%)
**Laboratory findings**		
LDL-C mmol/L, mean (IQR)	3.0 (2.3-3.7)	3.1 (2.4-3.9)
**Medication prior admission, n (%)**		
Aspirin	1,664 (18.3%)	22,595 (21.3%)
ACE-inhibitor or ARB	2,672 (29.5%)	29,408 (27.8%)
Beta blocker	2,180 (24.1%)	25,448 (24.1%)
Statin	1,646 (18.1%)	20,699 (19.5%)
**Initiated new prescription** ^*^ **, n (%)**		
Aspirin	6.076 (66.6%)	76.247 (71.2%)
ACEI/ARB	3.137 (34.4%)	54.398 (50.8%)
Beta blocker	4.915 (53.9%)	68.335 (63.9%)
Statin	5.739 (62.9%)	77.400 (72.3%)

ACEI/ARB, ACE-inhibitor or angiotensin-receptor blocker; BMI, body mass index.

* Patients without prior use of a particular drug group that were prescribed that drug group at discharge and initiated treatment within 30-days.

During follow-up, 1,914 MINOCA patients and 25,777 of MI-CAD patients suffered a MACE, including 865 and 11,656 all cause deaths, respectively. At total of 304 MINOCA patients and 5,343 MI-CAD patients suffered a CV-death.

### Persistence to secondary preventive medications and prognosis

**Fig 2:**
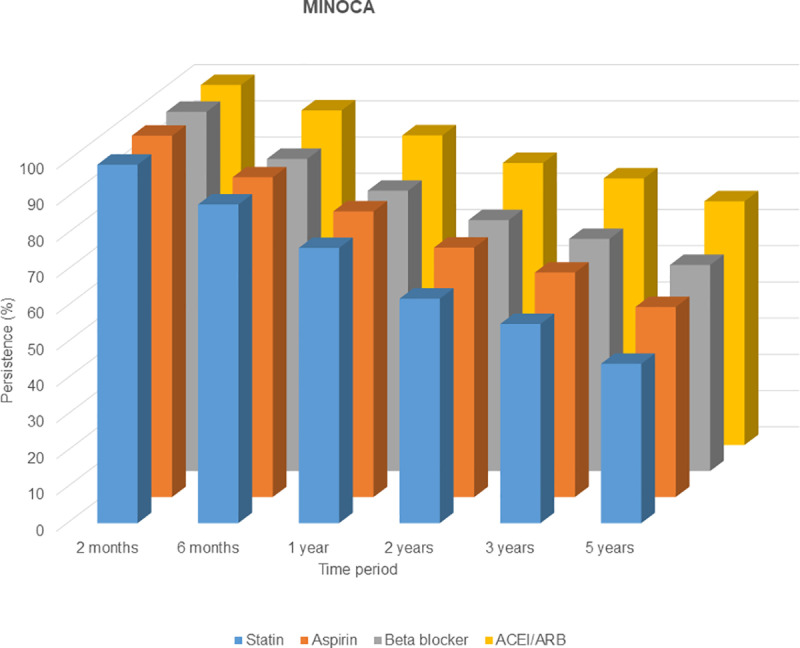
Persistence to secondary preventive medications at different time intervals for patients with MINOCA. The follow-up started at discharge and only patients who initiated drug were included in further analyses. The initial outpatient visit was after 6-8 weeks.

**Fig 3:**
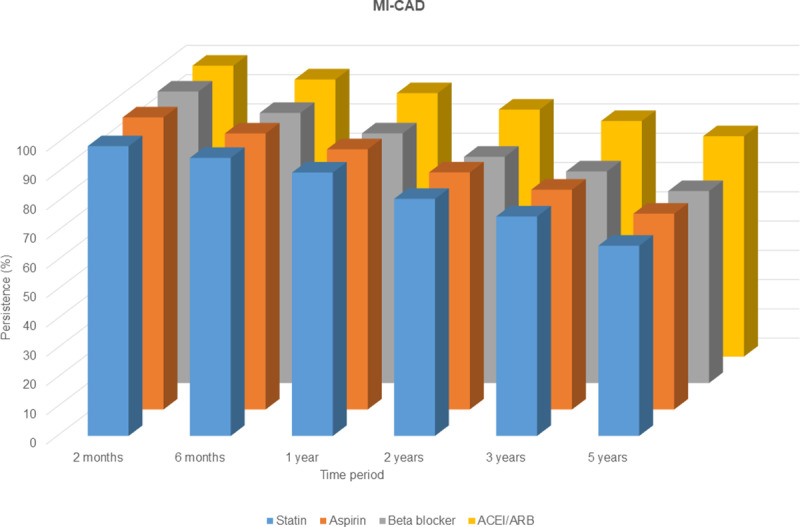
Persistence to secondary preventive medications at different time intervals for patients with MI-CAD. The follow-up started at discharge and only patients who initiated drug were included in further analyses. The initial outpatient visit was after 6-8 weeks.

Patients with MINOCA had lower persistence to all studied drug classes than patients with MI-CAD at all time periods. At 12 months, the persistence to statin was 76% vs. 90%, to aspirin 78.9% vs. 88.9%, to beta blockers 77.4% vs. 85.3% and ACEI/ARBs 85.5% vs. 90% in MINOCA and MI-CAD patients respectively (Figs 2 and 3) [21].

There was a strong covariation in the persistence to secondary preventive medications. A patient who was persistent with one secondary preventive drug was also more likely to be persistent with at least on more drug group (Table 2).

**Table 2:** The probability of patients being persistent to more than one secondary preventive drug. Presented as odds ratios for persistence to pairs of drugs at 12 months. All values are significant, p < 0.001.

	ACEI/ARB	Aspirin	Beta blocker	Statin
**MINOCA**				
ACEI/ARB		3.53	4.54	2.95
Aspirin			3.69	3.43
Beta blocker				2.77
Statin				
**MI-CAD**				
ACEI/ARB		3,19	3,84	2,91
Aspirin			2,96	3,36
Beta blocker				2,45
Statin				

ACEI/ARB, ACE-inhibitor or angiotensin-receptor blocker.

Persistent use of secondary preventive drugs in both patients with MINOCA and MI-CAD was associated with a decreased risk of MACE during follow-up (Table 3). The association between persistent use of secondary preventive drugs and all-cause death were strong in both MINOCA and MI-CAD patients (Table 4). The association between persistent use of all assessed drug-groups and CV death was also evident in MI-CAD patients, whereas only persistent use of aspirin was significantly associated with less CV-death in MINOCA patients (Table 4).

**Table 3:** The association between persistence of individual secondary preventive drugs and the risk for MACE in patients with MINOCA and MI-CAD.

	Endpoint	Drug	Person-time	Events^*^	Result, unadjusted^*^	Result, adjusted^†^	Results, corrected^
			1000 person-years		HR (95% CI)	HR (95% CI)	HR (98.75% CI)
**MINOCA**							
Persistent vs Non-persistent	MACE	ACEI/ARI	2.2/8.9	115/385	**0.69 (0.55 0.86)**	**0.62 (0.50-0.77)**	**0.62 (0.47-0.82)**
		Aspirin	5.1/16.1	244/601	**0.64 (0.55-0.76)**	**0.70 (0.60-0.82)**	**0.70 (0.57-0.86)**
		Beta blocker	5.2/12.6	216/522	0.88 (0.74-1.04)	**0.77 (0.65-0.92)**	**0.77 (0.62-0.96)**
		Statin	6.1/14.4	260/576	**0.78 (0.66-0.92)**	**0.80 (0.68-0.95)**	**0.80 (0.65-0.99)**
**MI-CAD**							
Persistent vs Non-persistent	MACE	ACEI/ARI	22.4/163.7	1,319/7,529	**0.63 (0.60-0.67)**	**0.67 (0.63-0.71)**	**0.67 (0.62-0.73)**
		Aspirin	32.4/229.7	2,141/9,284	**0.49 (0.46-0.51)**	**0.60 (0.57-0.64)**	**0.60 (0.57-0.64)**
		Beta blocker	46.0/189.7	2,144/8,540	**0.78 (0.74-0.82)**	**0.76 (0.73-0.80)**	**0.76 (0.72-0.81)**
		Statin	37.2/227.9	2,402/9,957	**0.52 (0.50-0.55)**	**0.66 (0.63-0.69)**	**0.66 (0.62-0.70)**

* Unexposed/exposed

† Adjusted for age, gender, bmi, index diabetes, index hypertension, index cholesterol level, index cancer, haemorrhagic stroke and bleeding

^Bonferroni-corrected for multiplicity within the set of four drugs

**Table 4:** The association between adherence of individual secondary preventive drugs and the risk for all-cause death and cardiovascular (CV) death in patients with MINOCA and MI-CAD.

	Endpoint	Drug	Person-time	Events	Result, unadjusted^*^	Result, adjusted^†^
			1000 person-years		HR (95% CI)	HR (95% CI)
**MINOCA**						
Persistent vs Non-persistent	Death	ACEI/ARI	2.4/9.4	90/187	**0.50 (0.38 -0.65)**	**0.43 (0.33 -0.57)**
		Aspirin	5.6/17.3	162/275	**0.52 (0.42 -0.64)**	**0.67 (0.54 -0.83)**
		Beta blocker	5.5/13.2	137/262	**0.80 (0.64 -0.99)**	**0.69 (0.56 -0.87)**
		Statin	6.5/15.1	166/267	**0.68 (0.55 -0.84)**	**0.74 (0.59 -0.91)**
	CV death	ACEI/ARI	2.1/8.6	25/68	0.66 (0.41 -1.06)	**0.60 (0.37-0.99)**
		Aspirin	4.9/15.8	56/90	**0.48 (0.33 -0.68)**	0.74 (0.51 -1.08)
		Beta blocker	4.9/12.1	46/90	0.80 (0.55 -1.17)	0.69 (0.47 -1.02)
		Statin	6.0/13.6	55/100	0.79 (0.55 -1.13)	0.95 (0.66 -1.38)
**MI-CAD**						
Persistent vs Non-persistent	Death	ACEI/ARI	24.8/175.3	374/1,650	**0.50 (0.47 -0.54)**	**0.59 (0.55 -0.63)**
		Aspirin	37.3/242.3	672/1,717	**0.40 (0.38 -0.43)**	**0.67 (0-63 -0.71)**
		Beta blocker	50.1/202.2	556/1,738	**0.74 (0.69 -0.79)**	**0.73 (0.69 -0.78)**
		Statin	41.6/242.4	745/1,944	**0.47 (0.44 -0.49)**	**0.70 (0.66 -0.74)**
	CV death	ACEI/ARI	21.5/158.3	292/1,377	**0.57 (0.50 -0.64)**	**0.69 (0.61 - 0.78)**
		Aspirin	32.5/218.7	504/1,623	**0.34 (0.31 -0.37)**	**0.65 (0.59 -0.72)**
		Beta blocker	44.1/183.1	381/1,628	**0.73 (0.66 -0.80)**	**0.73 (0.66 -0.81)**
		Statin	38.9/216.6	493/1,848	**0.43 (0.39 -0.47)**	**0.70 (0.64 -0.77)**

* Unexposed/exposed

† Adjusted for age, gender, bmi, index diabetes, index hypertension, index cholesterol level, index cancer, haemorrhagic stroke and bleeding.

Persistent and/or restarting use of secondary preventive medication (e.g., predefined “users”) was associated with decreased risk for MACE in patients with MI-CAD, while only use of aspirin and ACEI/ARB were significantly associated with decreased risk for patients with MINOCA (Table 5).

**Table 5:** The association between use of individual secondary preventive drugs part of the time and the risk for MACE in patients with MINOCA and MI-CAD.

	Endpoint	Drug	Person-time	Events^a^	Result, unadjusted^*^	Result, adjusted^†^
			**1000 person-years**		**HR (95% CI)**	**HR (95% CI)**
**MINOCA**						
**User vs Non-user**	**MACE**	**ACEI/ARI**	1.8/9.3	87/413	**0.76 (0.60-0.97)**	**0.65 (0.51-0.83)**
		**Aspirin**	3.5/18.3	168/677	**0.68 (0.57-0.81)**	**0.75 (0.63 -0.90)**
		**Beta blocker**	3.5/14.2	139/599	0.97 (0.80-1.18)	0.82 (0.68-1.00)
		**Statin**	4.2/16.3	175/661	0.84 (0.70-1.00)	0.84 (0.71-1.01)
**MI-CAD**						
**User vs Non-user**	**MACE**	**ACEI/ARI**	16.1/169.9	976/7,872	**0.63 (0.59-0.68)**	**0.67 (0.62-0.72)**
		**Aspirin**	17.6/244.5	1,408/10,017	**0.43 (0.40-0.45)**	**0.57 (0.54-0.61)**
		**Beta blocker**	26.4/209.3	1,368/9,316	**0.72 (0.68-0.76)**	**0.71 (0.66-0.75)**
		**Statin**	20.5/244.5	1,543/10,816	**0.48 (0.45-0.50)**	**0.60 (0.57-0.64)**

* Unexposed/exposed

† Adjusted for age, gender, bmi, index diabetes, index hypertension, index cholesterol level, index cancer, haemorrhagic stroke and bleeding.

### Persistence to secondary preventive medications and achievement of target range LDL-C at 12 months

Secondary preventive measures were collected from the 12-months follow-up reported in SWEDEHEART for a total of 3.535 MINOCA patients and 47.427 MI-CAD patients. The persistence to treatment with statins was strongly associated to the achievement of target range LDL-C (e.g., < 1.8 mmol/L), non-smoking and participation in exercise training within a cardiac rehabilitation program at 12 months in both patients with MINOCA and MI-CAD (Table 6). The association between persistence to treatment and a target range LDL-C was independent of LDL-C level at index hospitalization (Table 7).

**Table 6:** The association between the duration of the persistence to statin and the achievement of LDL-C < 1.8 mmol/L and secondary preventive lifestyle changes at 12 months in patients with MINOCA and MI-CAD. Only patients with a 12-months follow-up reported in SWEDEHEART was included in the analyses.

			Result, unadjusted*	Result, adjusted^†^	Results, unadjusted*	Result, adjusted^†^	Result, unadjusted*	Result, adjusted^†^
			OR (95% CI)	OR (95% CI)	OR (95% CI)	OR (95% CI)	OR (95% CI)	OR (95% CI)
	Persistence group	Patients (n)	**LDL**		**Smoking**		**Physical activity**	
**MINOCA**	<6 months	182	reference					
	6-12 months	263	**1.81 (1.02 -3.22)**	**1.97 (1.09 -3.55)**	**1.59 (0.86 -2.91)**	**1.47 (0.79 -2.74)**	**1.73 (1.18 -2.53)**	**1.67 (1.13 -2.46)**
	12 months	2,041	**6.42 (3.95 -10.43)**	**7.55 (4.57 -12.49)**	1.04 (0.66 -1.62)	0.99 (0.62 - 1.57)	**1.46 (1.07 - 1.99)**	**1.48 (1.08 -2.03)**
**MI-CAD**	<6 months	750	reference					
	6-12 months	1,361	0.99 (0.77-1.27)	1.02 (0.79 -1.32)	**1.40 (1.11 -1.76)**	**1.44 (1.13 -1.83)**	1.17 (0.98 -1.41)	1.18 (0.98 -1.41)
	12 months	33,266	**4.02 (3.28-4.92)**	**4.43 (3.61 -5.45)**	**1.62 (1.35 -1.94)**	**1.62 (1.34 -1.96)**	**1.54 (1.33 -1.78)**	**1.53 (1.32 -1.78)**

*Unexposed/exposed.

†Adjusted for age, gender, bmi, index diabetes, index hypertension, index cholesterol level, index cancer, haemorrhagic stroke and bleeding.

**Table 7:** Association between duration of persistence of statin and the achievement of target range LDL-C (<1.8 mmol/L) at 12-months in patients with different LDL-C at index hospitalization. Only patients with a 12-months follow-up reported in SWEDEHEART was included in the analyses.

			Result, unadjusted*	Result, adjusted^†^
			OR (95% CI)	OR (95% CI)
	Persistence group	Patients (n)		
**LDL > 3.0 mmol/L**				
MINOCA	<6 months	103	reference	reference
	6-12 months	141	2.28 (0.89 -5.83)	2.23 (0.86 -5.79)
	12 months	1,27	**9.30 (4.10-21.09)**	**9.24 (4.03 -21.16)**
MI-CAD	<6 months	384	reference	reference
	6-12 months	792	0.95 (0.65 -1.38)	1.00 (0.68 -1.46)
	12 months	22,262	**4.40 (3.20 - 6.05)**	**4.54 (3.30 -6.26)**
**LDL 1.8–3.0 mmol/L**				
MINOCA	<6 months	62	reference	reference
	6-12 months	109	1.58 (0.62 -4.01)	1.68 (0.65 -4.37)
	12 months	666	**6.66 (3.05 - 14.54)**	**7.18 (3.23 -15.95)**
MI-CAD	<6 months	304	reference	reference
	6-12 months	472	1.02 (0.70 -1.50)	1.02 (0.69 -1.49)
	12 months	9,649	**4.55 (3.28 -6.30)**	**4.66 (3.35 -6.48)**
**LDL < 1.8 mmol/L**				
MINOCA	<6 months	17	reference	reference
	6-12 months	13	3.31 (0.59 -18.57)	4.72 (0.67-33.34)
	12 months	105	**4.62 (1.44 -14.80)**	**7.59 (1.75 -32.86)**
MI-CAD	<6 months	62	reference	reference
	6-12 months	97	1.39 (0.60 -3.25)	1.30 (0.55 -3.05)
	12 months	1,355	**3.81 (1.83 -7.93)**	**4.01 (1.93 - 8.32)**

*Unexposed/exposed.

†Adjusted for age, gender, bmi, index diabetes, index hypertension, index cholesterol level, index cancer, haemorrhagic stroke and bleeding.

## Discussion

This large nationwide registry-based study demonstrated for the first time a strong association between the persistence to secondary preventive medication and prognosis in patients with MINOCA. The risk reducing effect is in the same magnitude for patients with MINOCA as for patients with MI-CAD, further strengthening the indications for secondary preventive medical treatment in MINOCA.

International guidelines from both the ESC [3] and AHA [2] have advocated the importance of secondary preventive medical treatment after MI, including aspirin, beta blockers, ACEI/ARI and statins. Since these guidelines mainly apply to patients with MI-CAD, the secondary preventive medical treatment of patients with MINOCA has been more arbitrary and dependent on individual physicians’ considerations. However, since 2019 there are recommendations from both ESC and AHA proposing MINOCA specific secondary preventive treatments with focus on reducing risk factors for cardiovascular disease [11,12].

In the present study persistent use of the secondary preventive medications aspirin, ACEI/ARB, beta blockers and statins, were associated with a decrease in the risk of the composite endpoint (MACE) in both MINOCA and MI-CAD patients. The results for MI-CAD is in concordance with several previous observational studies [4,5,8].

A strong covariation in persistence to one or more secondary preventive medications was demonstrated in the present study. To distinguish the prognostic effect of one individual group of drugs among the use of several others may therefore be challenging. Furthermore, patients who are persistent to secondary preventive drugs are also adherent to other secondary preventive interventions as well. In this study, patient with long-term persistence to statins tended to cease smoking, participate in physical activities and reach desired LDL-C goals at 12 months to a larger degree. This is in agreement with previous studies showing that patients initiating and adhering to secondary preventive treatments for lowering LDL-C and blood-pressure are more likely to engage in other health-promoting behaviors [23–25]. It is therefore always important to acknowledge the “healthy adherer” effect in studies evaluating the effects of therapies on health outcomes [26].

The differences between studies in the method used to measure persistence may make comparing results more complex [27]. The present study applied a strict initial definition, measuring persistence only in patients with primary adherence to treatment, and a less rigid follow-up approach including patients who restarted treatment in the user group. Both of these factors may have resulted in higher levels of persistence at later time points than observed with other approaches, but may better reflect real world conditions.

However, the results are in agreement with a previous review of five adjusted observational studies, which indicated survival benefits of statins, beta blockers and dual antiplatelet therapy and reduced risk for MACE of ACEI/ARB among patients with MINOCA [13]. Similar positive effects were found in a small observational study suggesting longer survival in MINOCA patients treated with statin and ACEI/ARB [16] and in large observational study indicating long-term beneficial effects of treatment with statins and ACEI/ARB on MACE in patients with MINOCA and a trend toward a positive effect of β-blocker treatment [15].

### Strengths and limitations

In this nationwide registry-based study, almost all patients hospitalized in Sweden for acute MI in 2006–2017 were included allowing analyses of large and unselected patient cohorts. The results reflect real-life practice as contrary to the setting of randomized controlled trials, thereby enhancing the generalizability. A nationwide registry with consecutive enrollment reduced the selection bias associated with studies of patients at selected hospitals or subscribed in a particular health care insurance system. In addition, restricting the assessment of persistence to patients who had a de novo prescription for a drug class of interest reduce the risk for prevalent user bias. Furthermore, as all patients evaluated in the present study were prescribed secondary preventive medications according to the SWEDEHEART register and collected the prescriptions within 30 days of discharge according to the ‘Prescribed drug register’, the likelihood of high quality of data increases. The Prescribed Drug register also has a high quality with more than 99.7% of all dispensed prescriptions in the country recorded with unique patient identifiers [18]. Such longitudinal dispensing databases are considered as the gold standard for studies assessing persistence [28].

The study has also some limitations. The analysis relied on ICD-codes and there is always a risk of coding errors, under- or over-reporting of some diagnoses. Data on dispensed drugs have previously shown to be valid, but there is no information on whether the patients actually consumed the medicines that were dispensed. We did not include information on multi-morbidity and socioeconomic status which have been assessed with both persistence and clinical outcomes, and thus acted as a confounder.

Furthermore, diagnostic criteria for MINOCA were proposed in 2017 making it impossible to determine how many patients, who today would meet the criteria for MINOCA, instead were diagnosed with a non-MI related condition during the study period [29]. With today’s increased availability to cardiac magnetic resonance imaging (MRI), had some patients likely been diagnosed with an alternative diagnose such as myocarditis and Takotsubo cardiomyopathy. Secondary preventive treatments specific for MINOCA were not proposed until year 2019 (AHA) and 2020 (ESC) and the prescription of secondary preventive drugs before then are unsystematic. However, the MINOCA patients evaluated in the present study were prescribed secondary preventive medications based on the assessment of the physician in charge of discharge.

## Conclusions

Persistence to secondary preventive medications after MI is associated with a reduction in the risk for MACE in both patients with MINOCA and MI-CAD. Continuous efforts to improve adherence to evidence-based medications in general to all patients with MI should be a priority.

## Supporting information

S1 Table Imputed variables and number of missing. For follow-up variables, only patients with a 12-month follow-up are included.
(DOCX)

## Abbreviations

ACEI: angiotensin-converting enzyme inhibitor
ARB: angiotensin-receptor blocker
MI-CAD: myocardial infarction with obstructive coronary arteries
MINOCA: myocardial infarction with non-obstructive coronary arteries
