# Supplementary material for: Impact of persistence to secondary preventive medication on prognosis for patients with myocardial infarction with and without obstructive coronary arteries
Source: PLoS One. 2025 May 29;20(5):e0324533. doi: 10.1371/journal.pone.0324533 (PMC12121825; doi:10.1371/journal.pone.0324533)
Supplement: S1 Table — For follow-up variables, only patients with a 12-month follow-up are included. (DOCX) [file pone.0324533.s001.docx]

**S1 Table 1. Imputed variables and number of missing.** For follow-up variables, only patients with a 12-month follow-up are included.

| Variable class | Patients (n) | Variable | Missing values, n (%) |
| --- | --- | --- | --- |
| Baseline variables | 116.143 | BMI | 12482 (10.7 %) |
|  |  | C-LDL | 26030 (22.4%) |
| Follow-up variables | 50.962 | Smoking | 76 (0.1%) |
|  |  | Systolic blood pressure | 5095 (10.0%) |
|  |  | C-LDL | 6280 (12.3%) |
|  |  | Physical activity | 113 (0.2%) |
| BMI, body mass index. C-LDL, low density lipoprotein cholesterol. | | | |
